# Opportunities to advance research, intervention, and policy on stigma, eating disorders, and body image

**DOI:** 10.1186/s40337-023-00857-1

**Published:** 2023-08-08

**Authors:** Rebecca L. Pearl, S. Bryn Austin, Hiba Jebeile, Laura D’Adamo, Denise E. Wilfley

**Affiliations:** 1 Department of Clinical and Health Psychology, University of Florida College of Public Health and Health Professions, Gainesville, FL USA; 2 Division of Adolescent and Young Adult Medicine, Boston Children’s Hospital, Boston, MA USA; 3 Children’s Hospital Westmead Clinical School, The University of Sydney, Westmead, Australia; 4 Department of Psychological & Brain Sciences and Center for Weight, Eating, and Lifestyle Science, Drexel University, Philadelphia, PA USA; 5 Department of Psychiatry, Washington University in St Louis School of Medicine, St Louis, MO USA

Stigma involves assigning labels and negative character traits (or stereotypes) to individuals who are viewed as “different.” This labeling is used to justify mistreatment and exert power over stigmatized individuals through diminished social status, discrimination, and overall devaluation as human beings [1]. Decades of research studies have documented stigmatization of people on the basis of body weight and other aspects of appearance. Weight stigma typically involves ascribing negative stereotypes to individuals with a high body weight or larger body [2]. Individuals with low body weight may also be viewed negatively and assumed to have eating disorders, which in turn are linked to stereotypes as well [3]. Stigmatization of persons with psychiatric disorders more generally can also extend to those with eating disorders. Due to misconceptions that body weight and eating disorder symptoms are entirely within an individual’s control, blame is rampant. This Special Issue aims to bring increased attention to stigma related to eating disorders, weight, and body image, including intersections with other forms of stigma, health impacts, and promising avenues for intervention.

## Identifying stigma at multiple levels

Negative beliefs and attitudes contribute to stigma at multiple levels. At the structural level of stigma, laws, policies, and cultural narratives (including in media) are created to produce societal conditions that foster unequal opportunities (or discrimination) and impair the well-being of stigmatized individuals [4]. For example, the lack of legislation aimed to protect against weight discrimination in most places in the world creates conditions in which individuals may be denied employment or promotion or unduly fired on the basis of their weight. Even when protections are present, subtle forms of bias and discrimination can still persist. Within institutions, body mass index (BMI) cutoffs are used to deny patients healthcare or to justify firing employees, including service members from positions in the military.

The interpersonal level of stigma includes teasing and bullying (especially among youth), criticism or other disparagement, discrimination, or physical threats or violence. Sources of interpersonal stigma can include family members, peers, educators, employers, service industry workers, healthcare providers, and strangers. For example, parents may criticize their child’s body size, and healthcare professionals (including in mental health settings) may convey frustration or blame toward patients struggling with eating disorders or refuse to treat them. Stigma in health care settings is quite common and particularly harmful, given that this is a vulnerable context for patients and one in which patients expect support to improve their health, not judgment and disparagement.

The intrapersonal level of stigma includes processes that occur within stigmatized individuals, such as anticipated and internalized stigma. For instance, individuals with a high weight may absorb negative societal messages and come to apply negative stereotypes to themselves, leading to low self-worth. As a result, they may also expect to be socially rejected by others.

Altogether, it is undeniable that stigma is a problem in relation to eating disorders, weight, and appearance. Still, estimates are lacking across diverse groups of the rates at which these types of stigmatizing experiences occur and the degree to which stigma is internalized. Documenting stigma experiences and internalization in clinical samples is needed to better understand the needs of patients seeking care for eating disorders, weight, or body image concerns. In addition, population-level data are needed to be able to track potential changes in stigma over time, especially in response to interventions.

## Health impacts

The adverse mental and physical health impacts of stigma are robustly documented and consistent across many different types of stigma, including due to eating disorders, weight, and other aspects of appearance. Negative weight-related comments from family members or teasing from peers during childhood and adolescence increase risk for poor body image and onset of eating disorders, among other psychological consequences [5]. These early encounters with weight stigma continue to impact individuals across the lifespan as well. Stigma operates as a form of chronic stress that causes psychological distress and impairments to inflammatory and immune responses in the body, increasing risk for health conditions such as cardiovascular disease [6]. Responses to stigma-induced stress often include behaviors that undermine health, such as disordered eating or substance use.

Stigma also damages health by affecting access to and quality of health care [7]. For example, patients with eating disorders may avoid seeking treatment due to concerns of being judged by healthcare providers (or due to past experiences of stigma in health care settings), and healthcare professionals may overlook eating disorder symptoms in patients with a high weight, male gender, or minoritized racial identity due to misconceptions about both weight and eating disorders. In addition, stigma and discrimination affect social determinants of health [8]: Unfairly denying someone employment on the basis of their weight will affect their financial resources, ability to live in a safe neighborhood, access to health insurance (in countries where insurance is primarily accessed through employment or private pay, such as the US), and other factors related to socioeconomic status that affect overall health and well-being.

Overall, evidence strongly supports connections between stigma and poor health. Still, much is unknown about specific psychological and biological mechanisms that explain these connections, and moderators that may account for the wide array of individual differences that appear in response to stigma. More research is also needed to understand how other forms of stigma, such as those targeting minoritized racial, ethnic, gender, or sexual orientation identities, intersect with stigma around weight and eating disorders. Furthermore, relatively little research has investigated how these other forms of stigma affect outcomes related to body image, weight, and eating disorders.

## The need for interventions

As researchers continue to gain a more comprehensive and mechanistic understanding of how structural, interpersonal, and intrapersonal stigma affects health, greater efforts are needed to develop and implement interventions that reduce stigma and its harmful effects. Including perspectives of people with diverse lived experience is key when designing and evaluating such interventions, to ensure that these interventions are meeting critical needs and are not inadvertently perpetuating stigma.

Interventions targeting stigma are needed at multiple levels, or may be multilevel in design [9]. For example, one intervention could target multiple levels of stigma by: changing policies within a healthcare system to address structural stigma; implementing trainings for healthcare providers and staff in the health system to reduce interpersonal stigma; and offering peer support groups for patients served by the health system to reduce internalized stigma. Other possible targets for stigma interventions include: anti-bullying and anti-discrimination laws; changing the public narrative through media representation, news coverage, and social media campaigns; and developing evidence-based psychological interventions to ease the burden of internalized stigma. Research that compares single-level versus multilevel interventions is needed to determine optimal approaches to reducing stigma. Testing strategies for wide-scale dissemination and implementation of interventions is as important as developing the interventions themselves. Each intervention may not be able to address all aspects of stigma, but researchers and clinicians must work together to ensure that support is available for individuals who experience stigma now, while continuing to advance long-term efforts to eradicate stigma from society.

Opportunities exist to learn from and form collaborations with experts in other areas of stigma to inform how to effectively intervene. Great strides have been made to reduce stigma due to HIV, mental illness, sexual orientation, and cancers. Studies are needed to test whether mechanisms of change for reducing other forms stigma operate similarly with eating disorder- and weight-based stigma, or whether unique mechanisms may be identified. Cross-disciplinary research that bridges knowledge across specialized and typically siloed fields of study has the potential to accelerate progress and proliferate benefits to far more people than any one research group or specialty could reach on its own. Such an approach could also produce interventions that are better able to address intersecting stigmas, rather than focusing on only one form of stigma at a time.

Finally, greater attention to stigma is warranted in existing interventions for eating disorders, weight, and body image concerns. This may include investigating whether aspects of current treatments, their delivery, or their scientific study (including lack of diverse representation in study samples and on research teams) may contribute to stereotypes and stigma, as well as how current treatments may exacerbate or mitigate stigma. For instance, one could investigate how eating disorder treatment affects experiences and internalization of weight stigma, and how these effects may differ by body size and changes in weight during treatment. Identifying ways to build upon existing treatments by adding elements that specifically target stigma, and testing the feasibility of this type of integrated approach, could also provide new effective and efficient methods for disseminating stigma interventions.

## Call for papers

We have launched this Special Issue in response to the recognized harms of structural, interpersonal, and intrapersonal levels of stigma and need for more ideas and evidence in this important area. We encourage papers that enhance understanding of stigma in relation to eating disorders, weight, or body image, with strong interest in studies that include diverse samples, examine intersecting forms of stigma, or analyze stigma at multiple levels. We also invite papers that contribute new knowledge of how stigma affects health and well-being, and that move toward developing and implementing innovative interventions to prevent and reduce stigma for people with eating disorders and body image concerns. Further invited areas include: intervention comparisons of single-level versus multi-level stigma-reducing interventions, identification of mechanisms of change of effective stigma reduction interventions, psychometric studies identifying reliable and valid measures of outcomes and change processes of stigma reduction programs, delineation of theoretical frameworks for topic-specific and/or general approaches to understand and reduce stigma, and implementation studies of evidence-based stigma reduction interventions. We encourage cross-disciplinary and multi-sector focused research that leverages varied perspectives and expertise in order to solve the complex problem of stigma.

## Data Availability

Not applicable.
